# Assessing the Relationship of APOE SNPs and Dementia in Native Hawaiians and Pacific Islanders

**DOI:** 10.1002/alz70855_103875

**Published:** 2025-12-24

**Authors:** Justina P Tavana, Maegan Leary, James E Galvin, JoAnn T. Tschanz, John Kauwe, Perry G Ridge

**Affiliations:** ^1^ Brigham Young University, Provo, UT, USA; ^2^ University of Miami Miller School of Medicine, Boca Raton, FL, USA; ^3^ Utah State University, Logan, UT, USA; ^4^ Brigham Young University‐Hawaii, Laie, HI, USA; ^5^ Brigham Young University, Laie, HI, USA

## Abstract

**Background:**

Alzheimer's disease (AD) is among the most significant public health and medical challenges of our day. Homogenous datasets limit the clinical utility of discoveries, possibly leading to population‐based disparities. Most AD research data were derived from White populations. Native Hawaiians and Pacific Islanders (NHPIs) have especially high risk: they have a higher AD to mild cognitive impairment ratio, earlier onset of AD and more cases of early‐onset AD, higher prevalence of female cases, more comorbidities that may contribute to AD, and worse MoCA/MMSE scores compared to Whites and Asians. NHPIs are the least represented minority group in large repositories/datasets. The NACC datasets consists of records for >50,000 subjects, of which fewer than 50 are NHPIs, and the AD Sequencing Project does not yet have any NHPI samples.

**Methods:**

We have recruited ∼2,000 older NHPIs through brain health fairs, in Utah, Hawaii, America Samoa, and the Kingdom of Tonga. Each participant provided a biospecimen and completed the AD8 to detect cognitive impairment. We genotyped the ε2 (rs7412) and ε4 (rs429358) APOE SNPs, compared SNP frequencies in NHPIs to frequencies in other known populations, and tested each for association with MCI. Each analysis was conducted using the aggregated dataset and separate NHPI populations.

**Results:**

Our cohort (to date, as additional collecting and genotyping is underway) consists of 1,367 older adults who self‐identify as NHPI. Our cohort includes 1,325 single‐ancestry participants (227 Native Hawaiians, 631 Samoans, 442 Tongans, and 25 others total from 8 different populations. The ε2 minor allele frequency (MAF; ∼7%) in NHPIs is comparable to other populations, whereas the ε4 MAF is approximately double (∼25%) in NHPIs compared to other known populations. However, neither SNP is correlated with MCI (*p*‐values ∼1).

**Conclusions:**

Neither the ε2 nor the ε4 APOE alleles predict MCI in NHPIs. Although our cohort is relatively small, we have sufficient power to detect effect sizes comparable to ε2 and ε4 effects in other populations. Research in other Indigenous populations has also failed to detect a significant correlation between APOE and dementia.

